# Trajectories in HbA1c and other risk factors among adults with type 1 diabetes by age at onset

**DOI:** 10.1136/bmjdrc-2021-002187

**Published:** 2021-05-31

**Authors:** Jon Edqvist, Araz Rawshani, Aidin Rawshani, Martin Adiels, Stefan Franzén, Lena Bjorck, Ann-Marie Svensson, Marcus Lind, Naveed Sattar, Annika Rosengren

**Affiliations:** 1 Department of Molecular and Clinical Medicine, Institute of Medicine, Gothenburg, Västra Götaland, Sweden; 2 Region Västra Götaland, Sahlgrenska University Hospital, Goteborg, Sweden; 3 Department of Public Health and Community Medicine, University of Gothenburg Health Metrics Unit, Gothenburg, Sweden; 4 National Diabetes Register, Centre of Registers, Gothenburg, Sweden; 5 Medicine, Uddevalla Hospital, Uddevalia, Region of Vastra Gotaland, Sweden; 6 BHF Glasgow Cardiovascular Research Centre, University of Glasgow, Glasgow, Scotland, UK

**Keywords:** diabetes mellitus, type 1, glycated hemoglobin A, cardiovascular system, albuminuria

## Abstract

**Introduction:**

In type 1 diabetes, potential loss of life-years is greatest in those who are youngest at the time of onset. Using data from a nationwide cohort of patients with type 1 diabetes, we aimed to study risk factor trajectories by age at diagnosis.

**Research design and methods:**

We stratified 30 005 patients with type 1 diabetes aged 18–75 years into categories based on age at onset: 0–10, 11–15, 16–20, 21–25, and 26–30 years. HbA1c, albuminuria, estimated glomerular filtration rate (eGFR), body mass index (BMI), low-denisty lipoprotein (LDL)-cholesterol, systolic blood pressure (SBP), and diastolic blood pressure trends were analyzed using mixed models. Variable importance for baseline HbA1c was analyzed using conditional random forest and gradient boosting machine approaches.

**Results:**

Individuals aged ≥16 years at onset displayed a relatively low mean HbA1c level (~55–57 mmol/mol) that gradually increased. In contrast, individuals diagnosed at ≤15 years old entered adulthood with a mean HbA1c of approximately 70 mmol/mol. For all groups, HbA1c levels stabilized at a mean of approximately 65 mmol/mol by about 40 years old. In patients who were young at the time of onset, albuminuria appeared at an earlier age, suggesting a more rapid decrease in eGFR, while there were no distinct differences in BMI, SBP, and LDL-cholesterol trajectories between groups. Low education, higher age, and poor risk factor control were associated with higher HbA1c levels.

**Conclusions:**

Young age at the diabetes onset plays a substantial role in subsequent glycemic control and the presence of albuminuria, where patients with early onset may accrue a substantial glycemic load during this period.

**Significance of this study**

**What is already known about this subject?**

- Patients with early onset of type 1 diabetes have a high excess risk of premature mortality, cardiovascular complications, and several life years lost, where poor glycemic control and adverse cardiovascular risk factors are associated with poor outcomes.
- Previous research has shown increased levels of HbA1c among patients with type 1 diabetes during adolescence and early adulthood; however, trajectories for glycemic controls and for other cardiovascular risk factors by age at onset has, to our knowledge, not been explored.

**What are the new findings?**

- Patients with early-onset type 1 diabetes (≤15 years) had a mean HbA1c of more than 70 mmol/mol in adolescence and early adulthood, whereas patients with later onset type 1 diabetes were approximately at target from the start, but then increased.
- By age 35 and later, these two categories had converged towards a mean of approximately ~60–65 mmol/mol.
- Early age at onset of type 1 diabetes was associated with higher probability of albuminuria earlier in life compared with patients with later onset.

**How might these results change the focus of research or clinical practice?**

- Our findings emphasize the importance of optimizing glycemic control in early-onset type 1 diabetes, in order to reduce lifetime glycemic load and to mitigate the gradual increase in HbA1c among patients with an onset later in life.
- The study also highlights the importance of multifaceted care for all patients with type 1 diabetes, potentially through increasing use of new technology, in order to reduce the probability of albuminuria, hypertension, hypercholesterolemia, and reduced kidney function.

## Introduction

Type 1 diabetes is associated with an increased risk of premature death, owing primarily to microvascular and macrovascular complications, which, in turn, are closely linked to risk factors such as glycemic control and renal function.^1–3^ Previously published data have shown that patients with type 1 diabetes have higher glycemic levels in adolescence or early adulthood than in childhood or later in adult life.^4^ This could partly explain the shorter life expectancy (by multiple years) in patients with early-onset type 1 as well as their markedly elevated risk of cardiovascular disease, which is thought to reflect an increased glycemic load over the life span in this group.^5^ Blood glucose within target has been observed to improve the excess risk in type 1 diabetes substantially,^2^ but to which extent this varies with age at onset has not been investigated. Thus, the first aim of this study was to investigate long-term differences in cardiovascular risk factor trajectories based on age at onset of type 1 diabetes. The second aim of this study was to identify predicting factors associated with baseline HbA1c based on age at onset among patients diagnosed with type 1 diabetes and with disease duration of at least 1 year.

## Research design and methods

### Study population

Sweden has a mainly publicly financed healthcare system, with low out-of-pocket costs for hospital visits and prescription drugs, and insulin and strips are free. Data were collected from the Swedish National Diabetes Registry (NDR) for patients enrolled from January 1, 1998 to December 31, 2012. The NDR is a nationwide registry, initiated in 1996, aimed at continuous improvement of management and validation of reported data^6^ and very high coverage, including virtually all Swedish patients diagnosed with type 1 diabetes.^2^ We defined patients with type 1 diabetes as those with a clinical diagnosis of type 1 diabetes provided by a physician, with an age of onset of ≤30 years and being treated with insulin. Information regarding cardiovascular disease, heart failure, chronic kidney disease, and amputations was retrieved using Swedish personal identification numbers linked onto the Swedish Hospital Registry (online supplemental table S1). Information about social factors—educational level, marital status, immigrant status, and annual income—was retrieved from the Longitudinal Integration Database For Health Insurance And Labour Market Studies (LISA) held by Statistics Sweden which is systematically collected for all Swedish citizens 16 years and older. Patients in NDR have given their written or verbal consent.

10.1136/bmjdrc-2021-002187.supp1 Supplementary data

The study group initially included 36 872 patients who attended 349 790 registered visits from 1998 to 2012. We excluded patients with a negative survival time (n=3); those with a clinically defined diabetes diagnosis other than type 1 (n=4286); patients or individual visits where the patient was older than 75 years of age (n=163); as well as those with significant severe microvascular complications (end-stage chronic renal disease (estimated glomerular filtration rate (eGFR) <15 or renal dialysis) or amputation) before their first registration (n=415). Because of partly incomplete data on severe retinopathy, we were unable to make this an exclusion criterion. After these exclusions, the final study group comprised 32 005 patients with 320 505 registered visits (see flowchart in online supplemental figure S1).

### Risk factors, descriptions of variables

Data on risk factors were obtained and reported to the NDR by physicians and nurses. HbA1c was initially measured with a Mono-s high-performance liquid chromatography system and converted into International Federation of Clinical Chemistry units (mmol/mol). The eGFR was calculated by the Modification of Diet in Renal Disease study equation, where serum creatinine is measured in μmol/L. For systolic blood pressure (SBP) and diastolic blood pressure(DBP), mm Hg was used, while low-denisty lipoprotein (LDL)-cholesterol was measured in mmol/L. Microalbuminuria and macroalbuminuria were defined as two out of three urine samples containing an albumin/creatinine ratio of 3–30 mg/mmol (~U-albumin of 20–200 µg/min (~20–300 mg/L)) or >30 mg/millimole (~U-albumin>200 µg/min (~>300 mg/L)), respectively.

### Mixed linear models and generalized mixed linear models

**Figure 1:**
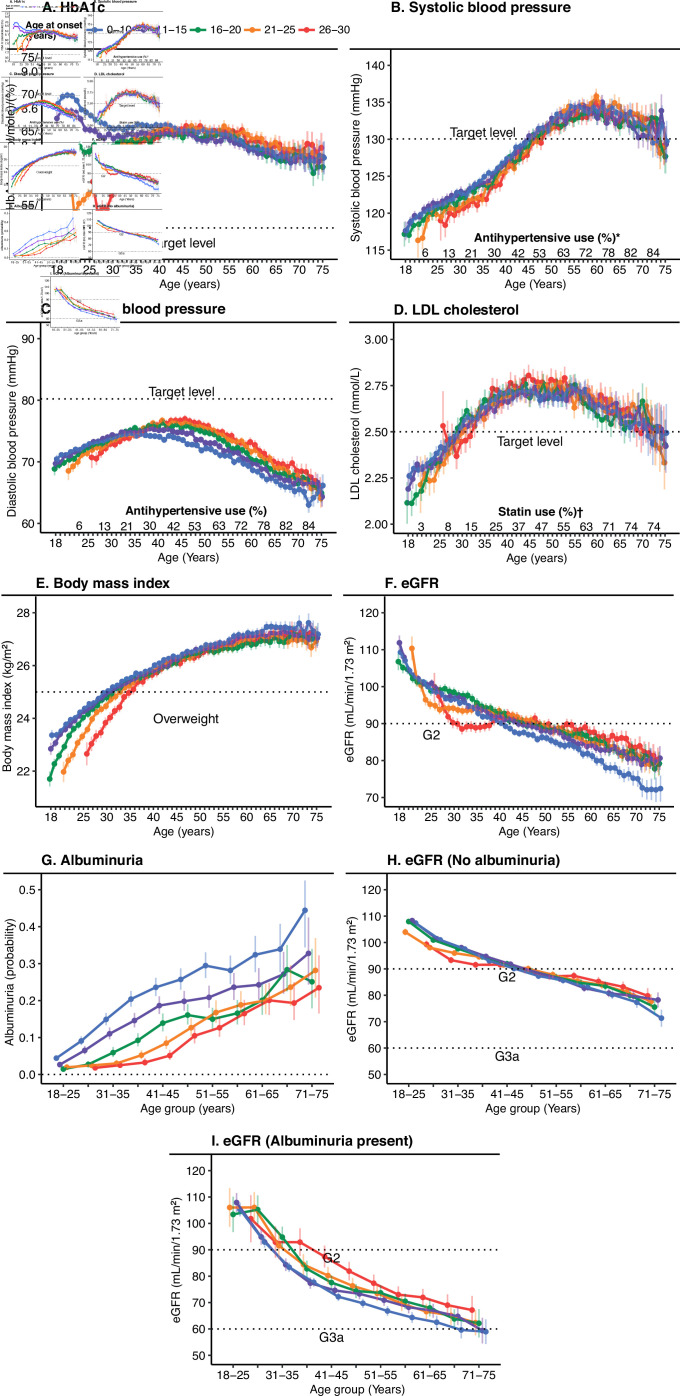
Trends for HbA1c and risk factors associated with type 1 diabetes in patients between 18 and 75 years of age stratified by age at onset of disease. Analyses were performed using mixed linear regression and generalized linear mixed models. Age, age at onset and the interaction between age and age at onset were set as fixed effects, with a random participant effect. Panels A–I were adjusted for sex. Panels B and C show additional adjustment for antihypertensives. Panel D shows additional adjustment for statin use. Panel E shows additional adjustment for smoking status. Panels A–F show analysis of each year between the ages of 18 and 75 years. Panels G–I show analysis of 4-year age intervals from 18 to 25 years of age to 71–75 years of age. *Proportion of the use of hypertensives calculated in 4-year age intervals from 18 to 25 years of age to 71–75 years of age. †Proportion of the use of statins calculated in 4-year age intervals from 18 to 25 years of age to 71–75 years of age. eGFR, estimated glomerular filtration rate; LDL, low-denisty lipoprotein.

To calculate risk factor trajectories (for HbA1c, SBP, LDL-cholesterol, and body mass index (BMI)), mixed linear regression was used and generalized linear mixed model for the outcome of albuminuria, where microalbuminuria and macroalbuminuria were combined into a binary outcome in order to increase power. The models were adjusted for sex, with an incorporated random participant effect, fixed effects of age, groups by age at onset (ie, defined as 0–10 years; 11–15 years; 16–20 years; 21–25 years; and 26–30 years), and an interaction term between groups by age at onset and age, where age was used as a categorical variable that allowed a separate estimate to be performed for each year (18–75 years of age) by the stratified groups by age at onset using least square means while albuminuria was back-transformed from the logit scale into probability. Antihypertensive medication (with SBP and DBP as outcomes), statin use (with LDL-cholesterol as outcome), and smoking status (with BMI as outcome) were added as adjustments to applicable models. In order to increase power in the analyses of albuminuria and eGFR subgroup analyses, which were stratified by the presence or absence of albuminuria, age was divided into age spans of 18–25, 26–30, 31–45, 46–50, 51–55, 56–60, 61–65, 66–70, and 71–75 years of age. The same age spans were used for calculations with respect to the proportions of statin use and antihypertensives presented in figure 1, counting users of the respective medications in each age interval, divided by the total number of patients in each age span, respectively. All sex specific models were performed separately.

### Baseline HbA1c and imputation of baseline predictors

To describe variables within the age at onset and sex groups, we used the first observed value (patients with recent-onset diabetes (<1 year from onset) were excluded from the analyses to eliminate unstable values occurring just after diagnosis) to obtain a cross-sectional picture of the study population, due to the large variation in terms of time to follow-up and number of visits. The patient cohort comprised patients aged 18–75 years with varying duration of diabetes at first inclusion in NDR. To identify variables associated with HbA1c level within the age at onset groups, we divided the patients by sex and age of onset of 0–15 years or 16–30 years. Missing values were imputed by Multiple Imputation by Chained Equations (MICE) using 10 iterations (R package MICE: V.3.11.0).^7^ Imputed data were used solely to provide a complete set of baseline data for the machine learning analysis. The imputed data were validated as shown in online supplemental figure S2, which presents the distribution of a subset of six of the 17 variables used, before and after imputation.

### Machine learning and prediction of baseline HbA1c levels

We used two machine learning algorithms, gradient boosting machine (GBM) and conditional random forest (CForest) (which uses two different algorithms^8–10^), to identify coexisting predictors for baseline HbA1c values stratified by age at onset and sex. All meaningful variables in the registry, that is, variables such as age, sex, socioeconomic status, and various cardiovascular risk factors, were analyzed with baseline HbA1c as the outcome. The results from the final predictions are shown as variable importance for HbA1c for an age at onset of 0–15 years or 16–30 years stratified by sex, generating a relative influence according to GBM that explains the relative contribution of the variables to the model. CForest output was measured by mean squared error, which was standardized into a percentage. Adjusted prediction figures generated by the models are shown in partial dependence plots created^11^ using R-package pdp (V.0.7.0) and 3D-interaction plots created using plotmo (V.3.5.7). Single partial dependence plots were created for the age variable to confirm consistency with the mixed linear models that were created using HbA1c level as the output. All statistical analyses were performed using R (V.3.4.3)

## Results

### Baseline and age characteristics

Baseline characteristics at the time of the first registration in the NDR are presented in table 1, stratified by age at onset. The age at first registration increased from 30.6 years among patient with onset at 0–10 years to 41.0 years in those first diagnosed at older than 25 years, with the proportion of men increasing from 50.2% to 59.5%. The duration of diabetes decreased from 24.3 to 13.6 years. No apparent differences were observed in total cholesterol and LDL cholesterol levels; however, SBP, BMI, frequency of statin use, and the proportion of smokers were slightly higher among patients aged 25–30 years at onset compared with patients who were younger at onset. In contrast, patients who were younger at the time of disease onset were more frequently on antihypertensives, had higher eGFR, and were more likely to be using insulin pumps. Sex-specific baseline data are presented in online supplemental table S2, which shows that the most notable differences between the sexes were the higher frequency of albuminuria and use of antihypertensives among men and the more frequent use of insulin pumps among women.

**Table 1:** Baseline characteristics among patients with type 1 diabetes by age at onset

	Patients, overall	0–10 years	11–15 years	16–20 years	21–25 years	25–30 years
Individuals, n	32 005	10 242	8164	5385	4722	3492
Women	14 341 (44.8)	5100 (49.8)	3719 (45.6)	2189 (40.6)	1919 (40.6)	1414 (40.5)
Age (years)	33.7 (13.6)	30.6 (12.7)	32.0 (13.5)	33.9 (14.3)	37.3 (13.4)	41.0 (12.2)
Diabetes duration (years)	18.9 (13.9)	24.3 (12.9)	19.1 (13.5)	16.0 (14.3)	14.3 (13.3)	13.6 (12.2)
Debut age of diabetes (years)	14.7 (7.5)	6.4 (2.7)	12.9 (1.4)	17.9 (1.4)	23.0 (1.4)	27.5 (1.1)
HbA1c (mmol/mol)/(%)	65.8 (15.7)/8.2 (1.4)	68.5 (15.0)/8.4 (1.4)	66.8 (15.1)/8.3 (1.4)	63.5 (15.8)/8.0 (1.5)	62.9 (16.4)/7.9 (1.5)	63.0 (16.3)/7.9 (1.5)
Total cholesterol (mmol/L)	4.7 (1.0)	4.7 (1.0)	4.7 (1.0)	4.6 (1.0)	4.7 (1.0)	4.8 (1.0)
LDL cholesterol (mmol/L)	2.6 (0.8)	2.6 (0.8)	2.6 (0.8)	2.6 (0.8)	2.7 (0.8)	2.8 (0.9)
Body mass index (kg/m**^2^**)	24.8 (3.7)	24.8 (3.6)	24.7 (3.7)	24.5 (3.8)	24.9 (3.9)	25.1 (3.8)
Systolic blood pressure (mm Hg)	125.4 (16.0)	124.5 (15.5)	125.1 (15.8)	125.4 (16.1)	126.5 (16.4)	127.1 (16.6)
Smokers	3945 (13.4)	1196 (12.6)	1012 (13.4)	610 (12.3)	633 (14.5)	494 (15.3)
*Albuminuria*						
No albuminuria	21 826 (85.2)	6681 (81.7)	5501 (84.7)	3817 (88.1)	3341 (87.9)	2486 (88.7)
Microalbuminuria	2298 (9.0)	907 (11.1)	586 (9.0)	314 (7.2)	284 (7.5)	207 (7.4)
Macroalbuminuria	1485 (5.8)	592 (7.2)	406 (6.3)	202 (4.7)	175 (4.6)	110 (3.9)
eGFR (mL/min/1.73 m2)	100.5 (28.3)	100.9 (28.8)	103.0 (29.1)	101.3 (28.5)	98.1 (26.8)	94.9 (25.1)
Antihypertensives	5630 (18.7)	1944 (20.3)	1442 (18.7)	860 (17.0)	774 (17.3)	610 (18.4)
Statins	2830 (9.7)	792 (8.5)	655 (8.8)	475 (9.6)	491 (11.3)	417 (13.0)
Insulin pump	2545 (17.4)	1377 (27.3)	775 (20.3)	208 (8.5)	108 (5.6)	77 (5.6)

Data are *n* (%) for categorical variables and mean (SD) for continuous variables unless otherwise indicated.

eGFR, estimated glomerular filtration rate; LDL, low-denisty lipoprotein.

### Risk factor trajectories

**Figure 2:**
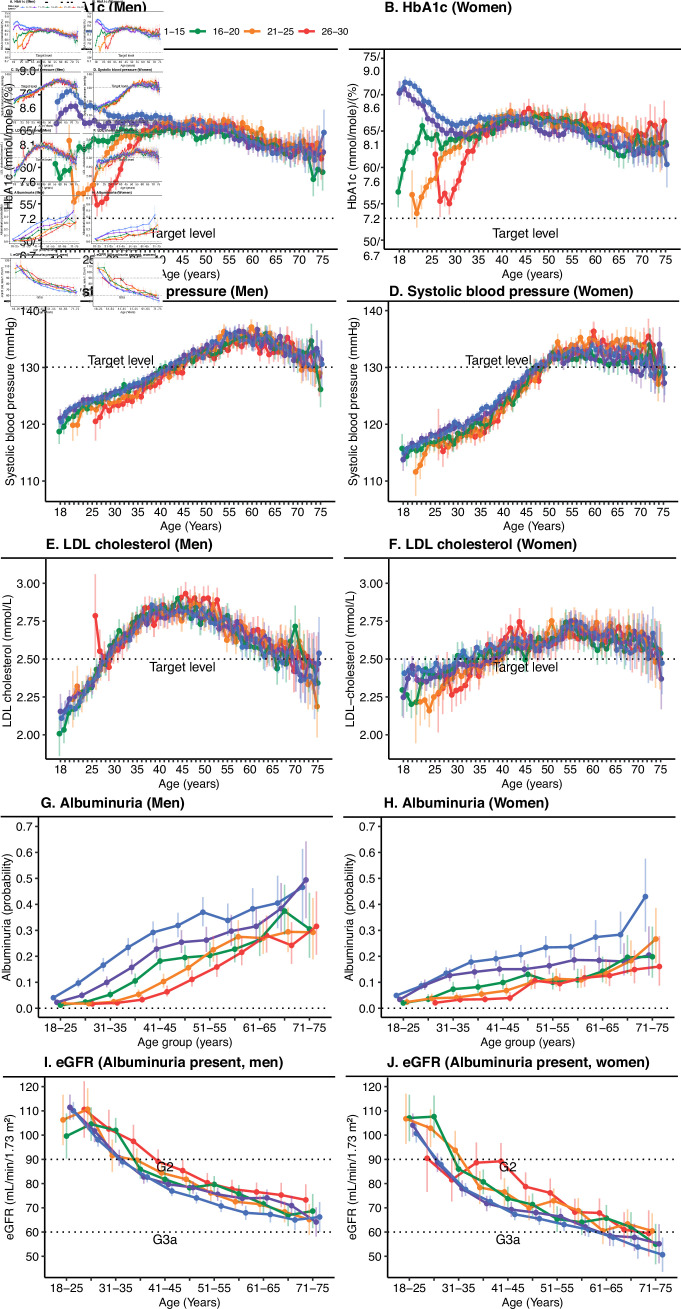
Trends for HbA1c and risk factors associated with type 1 diabetes in patients between 18 and 75 years of age stratified by age at onset of disease and sex. Analyses were performed using mixed linear regression and generalized linear mixed models. Age, age at onset and the interaction between age and age at onset were set as fixed effects, with a random participant effect. Panels C and D show additional adjustment for antihypertensives. Panels E and F show additional adjustment for statin use. Panels A–F show analysis of each year between the ages of 18 and 75 years. Panels G–J show analysis of 4-year age intervals from 18 to 25 years of age to 71–75 years of age. eGFR, estimated glomerular filtration rate; LDL, low-denisty lipoprotein.

The sex-adjusted mixed linear regression models displayed a substantial difference in HbA1c trends between ages 18 and 30 years (figure 1). Patients with disease onset at 15 years or younger entered adult life with a mean HbA1c of approximately 70 mmol/mol (8.6%), which leveled out after 30 years of age to approximately 65 mmol/mol (8.1%). Patients with an age at onset of 16 years or older instead experienced a gradual increase in HbA1c, with patients who experienced disease onset at 21–30 years of age exhibiting low initial mean HbA1c levels near target that subsequently increased to a mean of approximately 65 mmol/mol (8.1%) at about 40 years of age. The gradual increase in HbA1c level in the first years after diagnosis was even more prominent when patients who had had diabetes for less than 1 year were excluded (online supplemental figure S3). A similar pattern was observed among patients with age at onset 16–20 years, but with a more rapid increase in HbA1c levels that leveled off by approximately 25 years of age. All patients, regardless of age at onset, had a very similar, slightly downwards trajectory from early middle age until the age of 75 years, with fluctuating confidence intervals, although mean levels were relatively stable between approximately 60 (7.6%) and 65 mmol/mol (8.1%), regardless of age of onset (figure 1). There was a slightly steeper decline in eGFR with younger age at onset, where those with onset at 0–10 years had the lowest eGFR at 75 years of age, as well as by far the highest proportion of albuminuria at any age. The more pronounced fall in eGFR with age among patients with an early age at onset was only evident when albuminuria was already present. Changes in other risk factors including DBP, SBP, and LDL-cholesterol did not differ markedly by age at onset, increasing with age to reach a peak around age 55–60 years with a subsequent decrease for SBP, and a somewhat earlier peak for DBP and LDL-cholesterol. The proportion of statin use and antihypertensives increased gradually with age (figure 1). The trends stratified by sex (figure 2) showed very similar trajectories; however, women displayed slightly higher mean HbA1c levels, lower eGFR levels, and a flatter LDL-cholesterol curve than men, although men seemed to have a slightly higher risk of albuminuria in all age categories.

### Prediction models for baseline HbA1c levels based on age at onset

**Figure 3:**
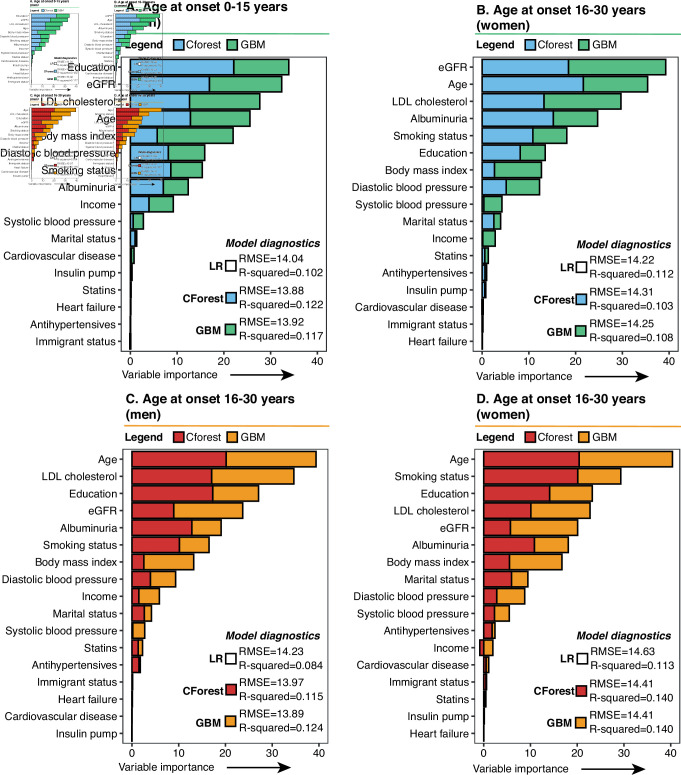
Variable importance for the first measured (baseline) stable HbA1c value (mmol/mol) in adulthood by age at onset and by sex. Analyses were performed using the conditional random forest and gradient boosting machine algorithms. Analyses included patients who had had a diabetes duration of 1 year or more. The y-axis shows the mean squared error output for the conditional random forest analysis standardized into a percentage and added to the relative influence output generated by the gradient boosting machine analysis. CForest, conditional random forest; eGFR, estimated glomerular filtration rate; GBM, generalized gradient boosting machine; LDL, low-denisty lipoprotein; LR, linear regression.

Imputed baseline characteristics by age of onset and by sex are presented in online supplemental table S3. The variable importance prediction for baseline (stable) HbA1c level based on GBM and CForest (figure 3) primarily highlighted eight predictors for all four models based on age at onset (0–15 years and 16–30 years at onset) and sex, that is, age, eGFR, albuminuria, blood pressure, LDL-cholesterol, smoking status, BMI, and educational level, although these predictors were ranked somewhat differently depending on age at onset or sex. Of note, the level of education achieved by adulthood was by far the dominant predictor for HbA1c levels in patients aged 15 years or younger at onset among men, while albuminuria was a more apparent predictor for HbA1c levels in women with a young age at onset than for men. In patients aged 16 years or older at the time of disease onset, smoking status among women was more dominant than for the other models, whereas age, LDL-cholesterol, and education were highlighted for men aged 16 years or older at onset. Our 3D plots confirmed that a low level of education, current smoking, high LDL cholesterol, low BMI, increased DBP, microalbuminuria or macroalbuminuria, and high eGFR were associated with higher levels of HbA1c, which is known clinically. However, interactions between, for instance, high DBP and high LDL cholesterol suggested that the levels of HbA1c seen in these patients may vary when these factors are combined (online supplemental figure S4). The single partial dependence plots created for age (online supplemental figure S5) suggested similar associations with respect to HbA1c levels as the mixed linear models presented in figure 1; and although no apparent interaction was observed, the model suggested that higher levels of education were associated with lower levels of HbA1c, regardless of age at onset and sex.

## Conclusion

In this nationwide study, we found substantial differences in mean HbA1c levels in patients younger than 35 years of age, depending on age at onset, such that onset before the age of 15 was associated with high HbA1c levels for a long period during early adulthood and an early increase in the probability of albuminuria that suggested a more rapid decline in eGFR. Later onset of disease was associated with initially low HbA1c levels that then increased gradually, as well as a slower increase in the probability of developing albuminuria than for patients with a young age of onset. After the age of 45, regardless of age at onset, patients with type 1 diabetes displayed stable and uniform mean HbA1c levels during the remaining observation period, declining slightly with age. Thus, in addition to a longer diabetes duration, an early age of onset was also associated with a substantially higher glycemic load up to the age of 35 years, suggesting that adolescence and early adulthood may be critical to improving outcomes in patients with type 1 diabetes. Mean LDL-cholesterol, SBP, and DBP levels were not affected by age at onset.

### HbA1c trajectories

We identified high mean HbA1c levels of approximately 70 mmol/mol (8.6%) in adolescent/young adult (18–25 years) patients who developed diabetes before 16 years of age that lasted for an extended period of time. This is a matter of considerable concern, because of the well-known risks of long-term complications such as cardiovascular disease and heart failure^2 12^ and effects on microvascular complications. Obviously, this could help explain the considerable loss of life-years experienced by this group of patients.^5^ The target for HbA1c levels is currently ≤52 mmol/mol (≤6.9%),^13 14^ and only an estimated 10%–15% of patients diagnosed before the age of 16 displayed a HbA1c within this target range in early adulthood, suggesting that treating glycemic levels as aggressively as possible in this group could reduce the risk of future complications. After the age of 30, HbA1c levels in all groups, regardless of age of onset, leveled off at the same average level of about 65 mmol/mol (8.1%), while the marked differences observed between the groups based on age at onset may be due to several causes.

Our study suggests that patients diagnosed in childhood are exposed to high glucose levels for a longer period compared with those who are diagnosed with type 1 diabetes in adolescence/adulthood. The HbA1c trajectories that we observed among patients with an age at onset of younger than 16 are very similar to those seen in a selected sample of predominantly privately insured US citizens with type 1 diabetes.^4^ The similarly poor glycemic control in early adulthood seen in both our study and the US study may be related to the transition between childhood and adulthood,^15 16^ where patients may sometimes experience a difficult transition from parental care onto independent management of their diabetes.^17^ Continuous glucose monitoring (CGM) has been shown to enhance glycemic control,^18^ although with an identified low patient adherence to sensor use among patients in aged 15–24 years compared with older individuals.^19^ Furthermore, hormonal changes along with social and psychological challenges in puberty may influence the ability to cope with diabetes care management in early adulthood.^20^

In contrast to patients with an early age at onset, patients with onset of diabetes at age 16 years or older exhibited substantially different HbA1c trajectories that were very similar to the trajectories of patients newly diagnosed with type 2 diabetes,^21^ that is, fast progression to elevated HbA1c levels, although we identified an even faster progression in HbA1c levels for individuals with a diagnosis of type 1 diabetes than the trends previously reported for patients with type 2 diabetes based on NDR data.^21^ Patients with an onset in adulthood have been observed to have low levels of HbA1c, which increased by time along with insulin doses, the first 5 years after diabetes onset.^22^ The present study showed that the increase in HbA1c could potentially last for >10 years, which could possibly reflect the increasing loss of beta cell mass which gradually decreases. The “honeymoon period” observed in patients with type 1 diabetes,^23^ possibly also applicable to patients with an onset in adulthood^24^ which might play a role. However, more research is needed on how to prolong, or keep the low levels of blood glucose observed the first ten years of follow-up. Why patients regardless of age at onset levelled off at a similar mean HbA1c in their 40s could be explained by that patients by then may have similar conditions in terms of insulin requirements and at a similar life phase, although reasons should be further investigated.

Importantly, inclusion in the current cohort was based on the clinician’s assessment of diabetes type, such that all patients included in our analyses were clinically diagnosed with type 1 diabetes. Nevertheless, our study suggests that all patients with type 1 diabetes should receive greater clinical attention and make use of modern treatment options. However, in many countries even basic levels of care in type 1 diabetes may be difficult to achieve.^25^

With increasing use of CGM and insulin pumps, metabolic control may improve but the extensive use of these devices in Sweden is fairly recent and therefore cannot yet be reliably assessed. Crude data from the 2017 NDR annual report suggest that the use of CGM increased from a mere 0.6% to almost 60% from 2015 to 2017, with recent findings suggesting improved glycemic control among pregnant women receiving CGM.^26^ The weak association between use of an insulin pump and glycemic control is most likely because insulin pumps are primarily used in patients with uncontrolled HbA1c levels.^27^ The long-term effects of CGM on HbA1c levels in patients with type 1 diabetes require further investigation; however, in agreement with guidelines, our study suggests that the use of medical technology is important for improving glycemic control in patients with type 1 diabetes.^28^

### Age at onset and risk factor control

Newly published research from the NDR suggests that patients with type 1 diabetes lose several life-years compared with the age-matched and sex-matched general population and that patients with a young age of onset have the worst prognosis and a markedly higher risk of cardiovascular complications and death.^5^ Lowering HbA1c may play a substantial role in decreasing the risk of death and death from cardiovascular complications where previous research has shown that a time-updated HbA1c within target was linked to a substantial reduction in mortality and death from cardiovascular causes.^2^ Whether lowering of glycemic load specifically for patients with an early onset of type 1 diabetes will have an impact on the excess risks of late complications and prolong life expectancy^5^ should be the subject of further investigation.

Other published data strongly emphasize the importance of controlling all risk factors, specifically HbA1c levels, LDL-cholesterol, blood pressure, smoking, and kidney function to reduce mortality and the incidence of cardiovascular complications.^29^ Additionally, patients with an early, as opposed to a late, onset are at markedly higher risk of having signs of renal dysfunction, further compounding the risk of early cardiovascular disease, heart failure, and death,^2 30^ and emphasizing the importance of caring for patients with a young age of onset to minimize the risk of late complications. Although there were few differences between the age at onset of 0–15 years and 16–30 years in terms of LDL-cholesterol, SBP, and DBP, generally increasing trends were observed until middle age. Our findings support findings that the traditional cardiovascular risk factors of LDL-cholesterol, SBP, and DBP should be optimized to lessen the overall risk factor burden and decrease the risk of cardiovascular outcomes,^1^ which may be of particular importance among patients with an early age of onset. However, this is a difficult balance to achieve, as younger people may be less inclined to take antihypertensive medications and statins, and some antihypertensives should be used with caution in women of childbearing age.

The foremost strength of the study is the nearly complete nationwide patient coverage afforded by the NDR, which enabled patients with prior cardiovascular disease and chronic kidney disease to be excluded from the analyses. Additionally, we had access to all individual visits from 1998 to 2012, which allowed analysis of repeated measurements, thus increasing the power of the estimates calculated for five different groups of patients aged between 18 and 75 years and stratified by age at onset. The primary limitation of the study is that it was conducted before CGM was implemented in Sweden. Even so, our results encourage the use of modern diabetes technology and are still relevant given that many adults even in high-income countries do not have access to all available technology due to healthcare costs. Additionally, our study did not have access to complete data from the day of onset in all patients registered, where some patients had a diabetes duration of several years before inclusion in the NDR. Also, we used no data on cardiovascular outcomes as this was outside the scope of the present study.

In conclusion, this study, which was conducted in the pre-CGM era, suggests that patients diagnosed early in life with type 1 diabetes generally have a high glycemic load, with substantial differences in HbA1c trajectories and the probability of developing albuminuria depending on the age at onset. Regardless of age at onset or sex, high HbA1c levels were predicted and coexisted with increased LDL cholesterol, DBP, and eGFR, presence of albuminuria, current smoking, and low educational level. This emphasizes the importance of multifaceted medical care, including use of modern technology, for patients with type 1 diabetes, with a focus on the need for optimizing glycemic control in patients with early-onset diabetes and attempting to mitigate the increase in HbA1c levels in patients with later-onset disease.

## Data Availability

Data may be obtained from a third party and are not publicly available. Data are available from the sources stated in the paper on request to the data providers, fulfilling legal and regulatory requirements and with permission from the Swedish Ethical Review Authority.
