# Supplementary material for: Trajectories in HbA1c and other risk factors among adults with type 1 diabetes by age at onset
Source: BMJ Open Diabetes Res Care. 2021 May 31;9(1):e002187. doi: 10.1136/bmjdrc-2021-002187 (PMC8169495; doi:10.1136/bmjdrc-2021-002187)
Supplement: Supplementary data [file bmjdrc-2021-002187supp001.pdf]

Trajectories in HbA1c and other risk factors among adults with type 1 diabetes by age at onset

Supplemental material

Supplementary table S1 - ICD-9 and ICD-10 codes for comorbidities at baseline

Diagnosis	Inpatient registry (ICD-9)	Inpatient registry (ICD-10)
Cardiovascular disease	431, 432X, 433, 434, 436, 437X	I21, I61, I62.9, I63, I64, I67.9
Heart failure	428	I50
Renal dialysis or transplantation	V42A, V45B, V56A, V56W	Z94.0, Z49, Z99.2
Amputation		NHQ09, NHQ11, NHQ12, NHQ13, NHQ14, NHQ16, NHQ17, NHQ99, NGQ09, NGQ19, NGQ99, NFQ19, NFQ99

Diagnosis used from the inpatient registry according to the International Classification of Diseases (ICD) system.

Supplementary table S2 - Baseline characteristics among patients with type 1 diabetes stratified by age at onset and by sex

	0-10 years (Men)	0-10 years (Women)	11-15 years (Men)	11-15 years (Women)	16-20 years (Men)	16-20 years (Women)	21-25 years (Men)	21-25 years (Women)	26-30 years (Men)	26-30 years (Women)
n	5142	5100	4445	3719	3196	2189	2803	1919	2078	1414
Women - n (%)	0 (0.0)	5100 (100.0)	0 (0.0)	3719 (100.0)	0 (0.0)	2189 (100.0)	0 (0.0)	1919 (100.0)	0 (0.0)	1414 (100.0)
Age (years)	30.5 (12.5)	30.8 (12.8)	31.3 (13.1)	32.8 (13.9)	33.3 (14.2)	34.8 (14.4)	36.7 (13.1)	38.2 (13.7)	40.6 (12.0)	41.7 (12.5)
Diabetes duration (years)	24.2 (12.8)	24.3 (13.0)	18.2 (13.2)	20.1 (13.8)	15.4 (14.1)	16.9 (14.5)	13.7 (13.0)	15.2 (13.6)	13.1 (11.9)	14.2 (12.5)
Debut age of diabetes (years)	6.3 (2.7)	6.5 (2.7)	13.1 (1.4)	12.7 (1.4)	17.9 (1.4)	17.9 (1.4)	23.0 (1.4)	23.0 (1.4)	27.5 (1.1)	27.5 (1.1)
HbA1c (mmol/mole)/(%)	68.2 (14.6)/8.4 (1.3)	68.8 (15.3)/8.4 (1.4)	66.6 (14.6)/8.2 (1.3)	67.1 (15.6)/8.3 (1.4)	63.0 (15.4)/7.9 (1.4)	64.3 (16.3)/8.0 (1.5)	62.7 (16.4)/7.9 (1.5)	63.3 (16.5)/7.9 (1.5)	62.5 (16.1)/7.9 (1.5)	63.6 (16.7)/8.0 (1.5)
Total cholesterol (mmol/L)	4.5 (1.0)	4.8 (1.0)	4.6 (1.0)	4.8 (1.0)	4.5 (1.0)	4.8 (0.9)	4.7 (1.0)	4.8 (1.0)	4.8 (1.0)	4.8 (1.0)
LDL cholesterol (mmol/L)	2.6 (0.8)	2.7 (0.8)	2.6 (0.8)	2.6 (0.8)	2.6 (0.8)	2.6 (0.8)	2.7 (0.8)	2.6 (0.8)	2.8 (0.9)	2.7 (0.8)
Body Mass Index (kg/m2)	24.6 (3.4)	24.9 (3.7)	24.7 (3.5)	24.7 (3.8)	24.6 (3.5)	24.5 (4.1)	25.0 (3.6)	24.6 (4.2)	25.2 (3.4)	25.0 (4.4)
Systolic blood pressure (mmHg)	126.8 (14.9)	122.1 (15.8)	126.9 (15.0)	123.0 (16.5)	127.1 (15.4)	123.0 (16.7)	128.1 (15.7)	124.1 (17.3)	128.0 (15.5)	125.6 (17.9)
Smokers - n (%)	514 (10.9)	682 (14.4)	484 (11.8)	528 (15.4)	315 (10.8)	295 (14.6)	348 (13.5)	285 (16.0)	268 (14.0)	226 (17.4)
Albuminuria - n (%)										
No albuminuria	3319 (80.8)	3362 (82.6)	2999 (84.4)	2502 (85.1)	2220 (86.9)	1597 (89.8)	1923 (86.0)	1418 (90.7)	1444 (87.3)	1042 (90.7)
Microalbuminuria	473 (11.5)	434 (10.7)	323 (9.1)	263 (8.9)	201 (7.9)	113 (6.4)	185 (8.3)	99 (6.3)	141 (8.5)	66 (5.7)
Macroalbuminuria	316 (7.7)	276 (6.8)	231 (6.5)	175 (6.0)	134 (5.2)	68 (3.8)	128 (5.7)	47 (3.0)	69 (4.2)	41 (3.6)
eGFR (mL/min/1.73m^2)	104.6 (28.4)	97.0 (28.8)	107.3 (27.8)	97.7 (29.7)	105.2 (28.3)	95.5 (27.9)	100.7 (26.0)	94.1 (27.5)	98.0 (24.8)	90.2 (24.9)
Antihypertensives - n (%)	1020 (21.2)	924 (19.3)	815 (19.5)	627 (17.9)	537 (17.9)	323 (15.7)	479 (18.0)	295 (16.2)	361 (18.3)	249 (18.6)

Statins - n (%)	413 (8.8)	379 (8.2)	356 (8.8)	299 (8.8)	279 (9.5)	196 (9.8)	309 (12.0)	182 (10.4)	260 (13.6)	157 (12.1)
Insulin pump	639 (24.7)	738 (30.1)	413 (19.1)	362 (21.8)	109 (7.3)	99 (10.3)	52 (4.4)	56 (7.5)	35 (4.2)	42 (7.7)
Treatment: Insulin	5142 (100.0)	5100 (100.0)	4445 (100.0)	3719 (100.0)	3196 (100.0)	2189 (100.0)	2803 (100.0)	1919 (100.0)	2078 (100.0)	1414 (100.0)

Data are *n* (%) for categorical variables and mean (SD) for continuous variables unless otherwise indicated.

Supplementary table S3 – Imputed baseline characteristics among patients with a diabetes duration >1 year from onset, stratified by age at onset and by sex

	0-15 years (Men)	0-15 years (Women)	16-30 years (Men)	16-30 years (Women)
n	9581	8811	7194	5015
Age (years)	30.9 (12.8)	31.6 (13.3)	38.0 (13.5)	39.2 (13.8)
Marital status - n (%)				
Married	1961 (20.5)	2214 (25.1)	2563 (35.6)	2101 (41.9)
Single	7156 (74.7)	5925 (67.2)	4022 (55.9)	2297 (45.8)
Divorced	437 (4.6)	591 (6.7)	570 (7.9)	527 (10.5)
Widowed	27 (0.3)	81 (0.9)	39 (0.5)	90 (1.8)
Education - n (%)				
9 years or less	2200 (23.0)	1812 (20.6)	1446 (20.1)	944 (18.8)
10 to 12 years	5410 (56.5)	4842 (55.0)	3793 (52.7)	2551 (50.9)
College or university	1971 (20.6)	2157 (24.5)	1955 (27.2)	1520 (30.3)
Income (hundreds, SEK)				
Q1	2417 (25.2)	2227 (25.3)	829 (11.5)	652 (13.0)
Q2	1657 (17.3)	2295 (26.0)	1000 (13.9)	1168 (23.3)
Q3	1606 (16.8)	2017 (22.9)	1265 (17.6)	1236 (24.6)
Q4	1889 (19.7)	1332 (15.1)	1763 (24.5)	1130 (22.5)
Q5	2012 (21.0)	940 (10.7)	2337 (32.5)	829 (16.5)
Swedish born - n (%)	9298 (97.0)	8488 (96.3)	6816 (94.7)	4702 (93.8)
Cardiovascular disease	236 (2.5)	194 (2.2)	214 (3.0)	131 (2.6)
Heart failure	58 (0.6)	68 (0.8)	55 (0.8)	41 (0.8)
Diabetes duration (years)	21.4 (13.3)	22.5 (13.5)	15.9 (13.0)	17.2 (13.4)
Age at onset	9.4 (4.1)	9.1 (3.8)	22.1 (4.1)	22.0 (4.1)
HbA1c (mmol/mole)	67.5 (14.7)	68.1 (15.4)	62.8 (15.0)	64.0 (15.8)
LDL cholesterol (mmol/L)	2.6 (0.8)	2.7 (0.8)	2.7 (0.8)	2.7 (0.8)
Body Mass Index (kg/m^2)	24.6 (3.5)	24.8 (3.8)	25.0 (3.5)	24.8 (4.1)
Systolic blood pressure (mmHg)	126.6 (14.9)	122.3 (16.0)	128.6 (15.7)	124.7 (17.2)
Diastolic blood pressure (mmHg)	73.5 (9.1)	71.8 (8.7)	74.8 (9.0)	72.8 (8.6)
Smokers - n (%)	1078 (11.3)	1320 (15.0)	942 (13.1)	817 (16.3)
Albuminuria - n (%)				
No albuminuria	7962 (83.1)	7436 (84.4)	6193 (86.1)	4495 (89.6)
Microalbuminuria	978 (10.2)	863 (9.8)	620 (8.6)	331 (6.6)
Macroalbuminuria	641 (6.7)	512 (5.8)	381 (5.3)	189 (3.8)
eGFR (mL/min/1.73 m^2)	98.3 (26.4)	89.5 (27.2)	92.4 (24.4)	84.4 (24.4)
Antihypertensives - n (%)	1896 (19.8)	1604 (18.2)	1415 (19.7)	887 (17.7)

Statins - n (%)	829 (8.7)	727 (8.3)	897 (12.5)	570 (11.4)
Insulin pump	1051 (11.0)	1099 (12.5)	193 (2.7)	193 (3.8)

Data are *n* (%) for categorical variables and mean (SD) for continuous variables unless otherwise indicated.

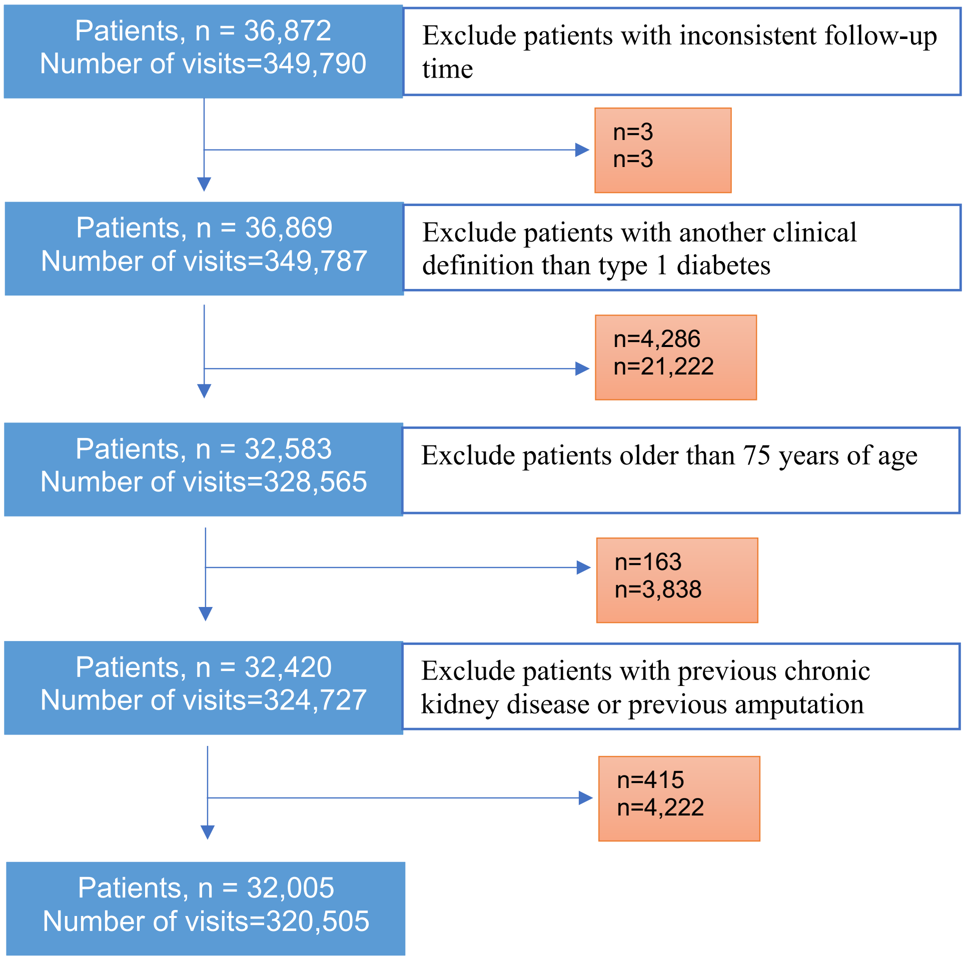

Supplementary Figure S1

Flow chart

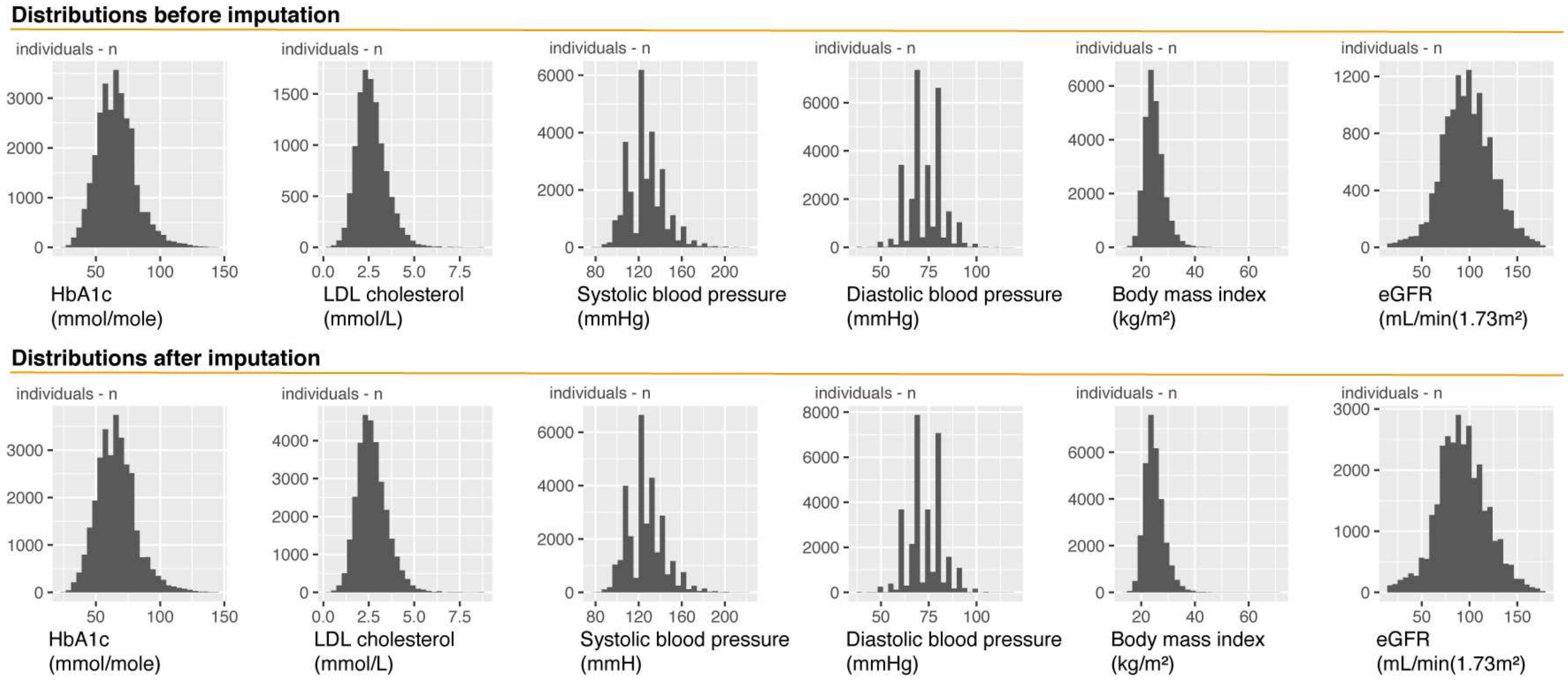

Supplementary Figure S2

Subample of variables describing distributions before and post imputation of baseline variables.

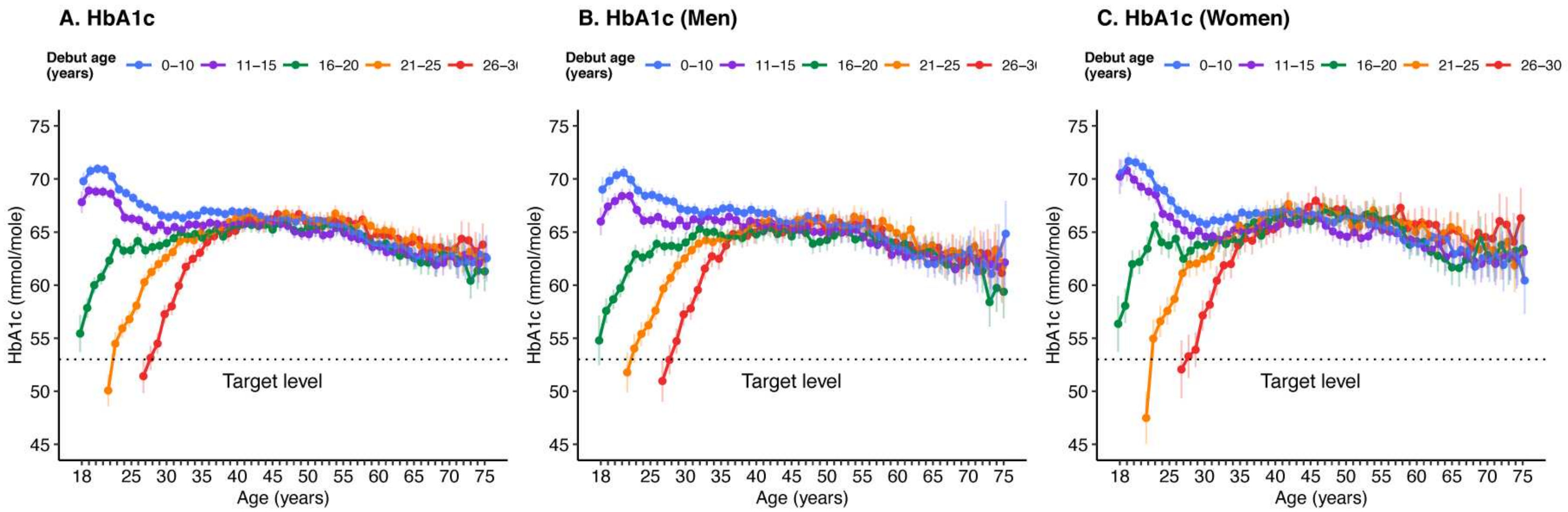

Supplementary Figure S3 - Trends for HbA1c associated with type 1 diabetes between 18-75 years of age stratified by age at onset and sex

Analyses were performed with Mixed Linear Regression and Generalized linear mixed model. Age, age at onset and the interaction between age and age at onset were set as fixed effects, with a random participant effect. Panel A was adjusted for sex.

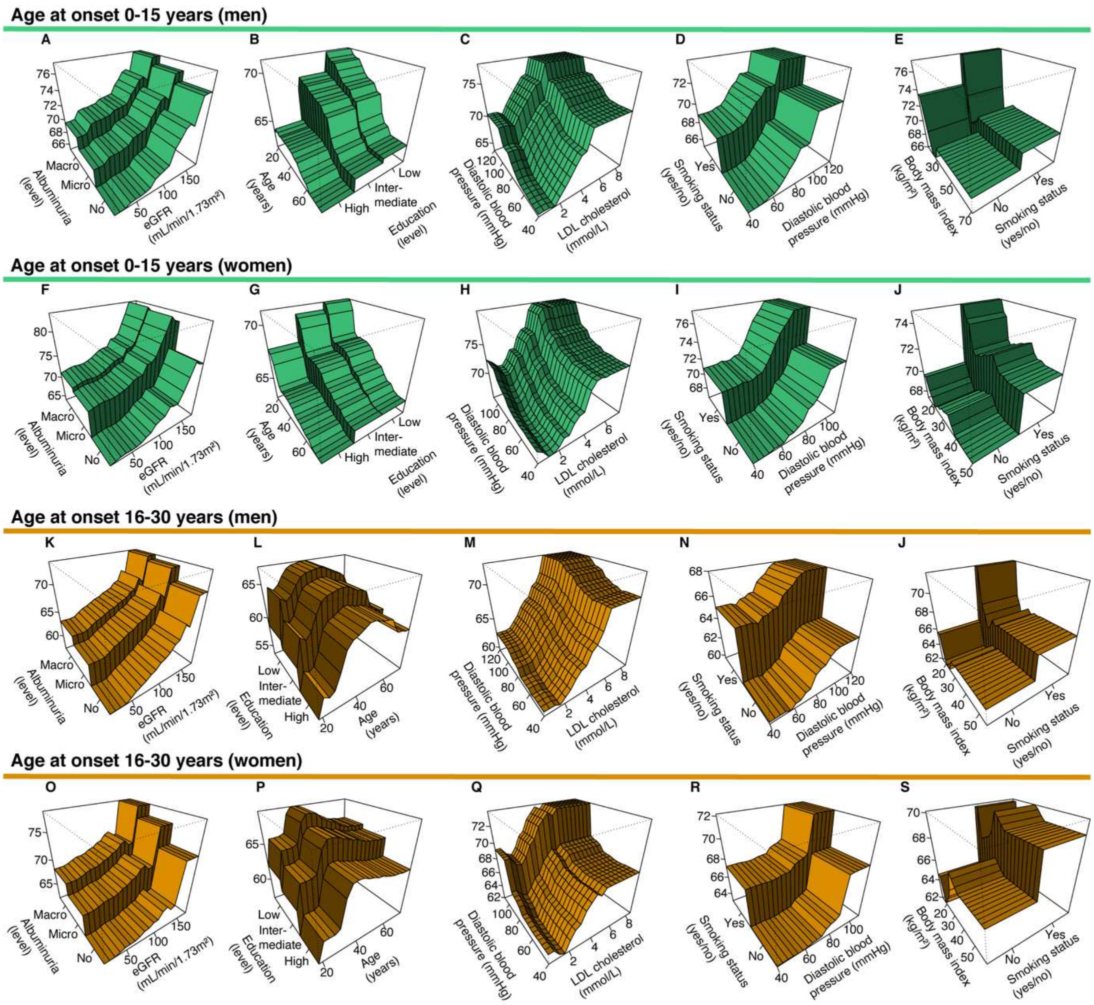

Supplementry Figure S4 - Partial dependence plots, showing interaction effects for the most important variables presented in Figure 3

3D partial dependence plots showing the adjusted average target response from the top predictors. Plots were generated from the Gradient boosting machines prediction presented in Figure 3. Panels A-E, F-J, K-J and O-S contains interactions of features in following order: albuminuria*eGFR; age*education; diastolic blood pressure*LDL cholesterol; smoking status*diastolic blood pressure; body mass index*smoking status.

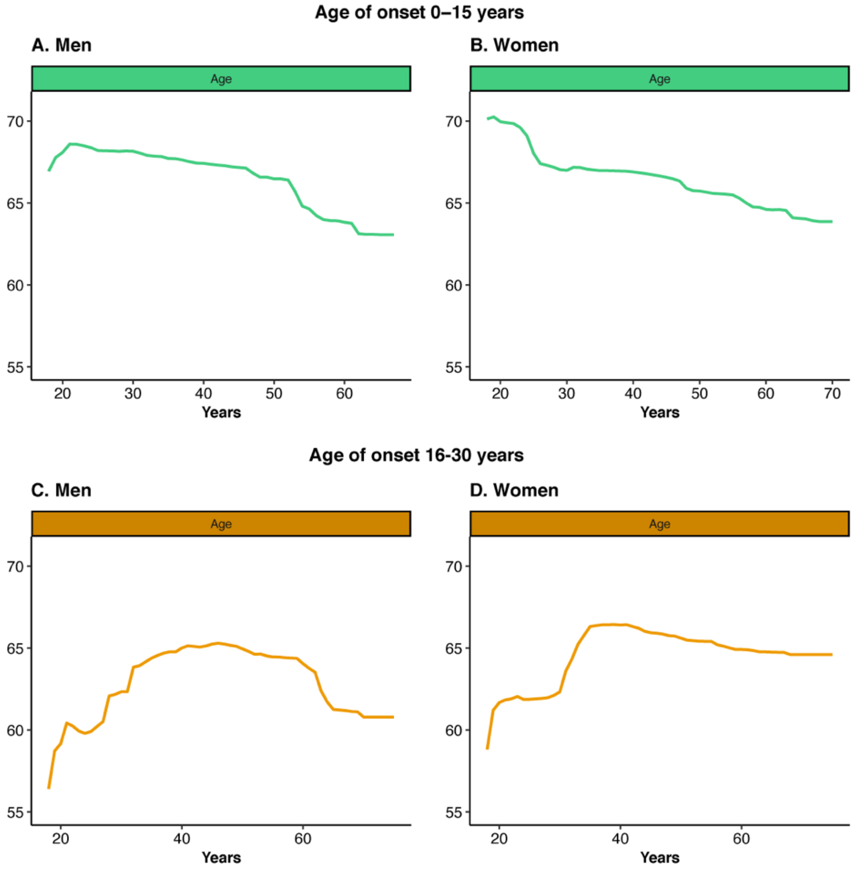

Supplementary Figure S5 – Single Partial dependence plot containing age based on GBM models presented in Figure 3

Single partial dependence plots showing the adjusted average target response from age. Plots were generated from the Gradient boosting machines prediction presented in Figure 3.
